# Chemokine receptor expression by inflammatory T cells in EAE

**DOI:** 10.3389/fncel.2014.00187

**Published:** 2014-07-04

**Authors:** Jyothi Thyagabhavan Mony, Reza Khorooshi, Trevor Owens

**Affiliations:** Neurobiology Research, Institute of Molecular Medicine, University of Southern DenmarkOdense, Denmark

**Keywords:** mouse, EAE, T cell, chemokine receptor, cytokine

## Abstract

Chemokines direct cellular infiltration to tissues, and their receptors and signaling pathways represent targets for therapy in diseases such as multiple sclerosis (MS). The chemokine CCL20 is expressed in choroid plexus, a site of entry of T cells to the central nervous system (CNS). The CCL20 receptor CCR6 has been reported to be selectively expressed by CD4^+^ T cells that produce the cytokine IL-17 (Th17 cells). Th17 cells and interferon-gamma (IFNγ)-producing Th1 cells are implicated in induction of MS and its animal model experimental autoimmune encephalomyelitis (EAE). We have assessed whether CCR6 identifies specific inflammatory T cell subsets in EAE. Our approach was to induce EAE, and then examine chemokine receptor expression by cytokine-producing T cells sorted from CNS at peak disease. About 7% of CNS-infiltrating CD4^+^ T cells produced IFNγ in flow cytometric cytokine assays, whereas less than 1% produced IL-17. About 1% of CD4^+^ T cells produced both cytokines. CCR6 was expressed by Th1, Th1+17 and by Th17 cells, but not by CD8^+^ T cells. CD8^+^ T cells expressed CXCR3, which was also expressed by CD4^+^ T cells, with no correlation to cytokine profile. Messenger RNA for IFNγ, IL-17A, and the Th1 and Th17-associated transcription factors T-bet and RORγt was detected in both CCR6^+^ and CXCR3^+^ CD4^+^ T cells. IFNγ, but not IL-17A mRNA expression was detected in CD8^+^ T cells in CNS. CCR6 and CD4 were co-localized in spinal cord infiltrates by double immunofluorescence. Consistent with flow cytometry data some but not all CD4^+^ T cells expressed CCR6 within infiltrates. CD4-negative CCR6^+^ cells included macrophage/microglial cells. Thus we have for the first time directly studied CD4^+^ and CD8^+^ T cells in the CNS of mice with peak EAE, and determined IFNγ and IL17 expression by cells expressing CCR6 and CXCR3. We show that neither CCR6 or CXCR3 align with CD4 T cell subsets, and Th1 or mixed Th1+17 predominate in EAE.

## Introduction

Multiple sclerosis (MS) is an inflammatory demyelinating disease of the central nervous system (CNS) whose pathogenesis involves infiltrating immune cells, including T cells. CD4^+^ T cells play a central role in orchestrating immune responses by secreting cytokines that regulate various cellular functions. Effector CD4^+^ T cells of Th1 and Th17 subsets are found in MS lesion and can mediate experimental autoimmune encephalomyelitis (EAE), an animal model of MS. Expression of Th1 and Th17 cytokines, IFNγ and IL-17 is detected in MS lesions (Steinman, 2008). EAE can be induced by the adoptive transfer of CNS antigen reactive Th1 cells (Pettinelli and Mcfarlin, 1981; Ando et al., 1989; Merrill et al., 1992; Baron et al., 1993) and Th17 cells (Langrish et al., 2005; Jäger et al., 2009; Domingues et al., 2010). While EAE induced by adoptive transfer of Th1 cells is characterized by infiltrates predominantly comprising of macrophages, EAE induced by Th17 cells is characterized by neutrophil recruitment (Kroenke et al., 2008).

MHC-I restricted CD8^+^ T cells are also suggested to play pathogenic roles in MS and its different animal models (Huseby et al., 2012). CD8^+^ T cells are present in the immune infiltrates in MS lesions (Traugott et al., 1983). CD8^+^ T cells in MS lesions are oligoclonally expanded (Babbe et al., 2000) and outnumber CD4^+^ T cells as the most frequent T cell subset in MS lesions (Hauser et al., 1986; Babbe et al., 2000). MHC-I molecules that present antigens to CD8^+^ T cells are highly expressed in astrocytes, oligodendrocytes, and neurons (axons) within the MS lesions suggesting that CD8^+^ T cells can directly engage these cells (Höftberger et al., 2004).

Migration of activated T cells into the CNS is directed by chemokines (Holman et al., 2011) and mediated by adhesion molecules (Engelhardt and Ransohoff, 2012). Constitutive expression of the chemokine CCL20 in choroid plexus is proposed to act as a gateway for T cells into uninflamed CNS (Axtell and Steinman, 2009; Reboldi et al., 2009). Th17 cells can preferentially express CCR6, the chemokine receptor for CCL20, *in vitro* (Hirota et al., 2007; Pötzl et al., 2008; Singh et al., 2008; Yamazaki et al., 2008; Reboldi et al., 2009). Based on the preferential expression of CCR6 in Th17, constitutive expression of CCL20 in choroid plexus and the requirement of CCR6 expression in CD4^+^ T cells for EAE, it is proposed that CCR6 plays a critical role in the entry of Th17 cells into the CNS in EAE and in induction of disease (Reboldi et al., 2009). The chemokine receptor CXCR3 binds CXC chemokines such as CXCL10 and is also of interest in EAE, although consensus is lacking on its precise role (Liu et al., 2005; Muller et al., 2010).

Forced expression of RORγt, the transcription factor critical for Th17 differentiation, can result in CCR6 expression (Ivanov et al., 2006; Hirota et al., 2007). However, RORγt expression in CD4^+^ T cells does not guarantee CCR6 expression *in vivo*. Although CCR6 expression correlates well with RORγt expressing IL-17 producers, CD4^+^ T cells that do not produce IL-17 can also express CCR6 (Wang et al., 2009). It is not known whether Th1 cells *in vivo* can also express CCR6.

We have assessed whether CCR6 identifies specific inflammatory T cell subsets in the CNS of mice with EAE, by direct analysis of CNS-infiltrating cells, with minimal manipulation. We find that Th1 outnumber Th17 CD4^+^ T cells, and that CCR6 is expressed by both, as well as by Th1+17. We also show that CD8^+^ T cells express CXCR3 rather than CCR6, and do not express IL-17. Thus chemokine receptors do not align with cytokine profiles amongst CNS-infiltrating T cells.

## Materials and methods

### Animals

C57BL/6 (B6) female mice were purchased from Taconic (Ry, Denmark). Mice were provided with food and water *ad libitum*. The mice were allowed to acclimatize with the environment in animal facility for a week before immunization. The experiments were carried out in accordance with rules and regulations laid down by Danish Justice Ministry Committee on Animal Research (Approval Number 2012-15-2934-00110).

### EAE

Mice were immunized by subcutaneous injection of 100 μl emulsion (50 μl on each side) containing myelin oligodendrocyte glycoprotein (MOG) p35-55 (100 μg) and complete Freund’s adjuvant (CFA) with heat inactivated *Mycobacterium tuberculosis* H37RA (200 μg; Difco Laboratories, Detroit) in the inguinal region. Animals received an intraperitoneal injection (200 μl) of pertussis toxin (0.3 μg; Sigma-Aldrich, Brøndby, Denmark) at the time of immunization and 2 days post-immunization (dpi). MOG p35-55 was synthesized at the Center for Experimental Bioinformatics (CEBI), Department of Biochemistry and Molecular Biology, University of Southern Denmark.

Mice were monitored for loss of body weight and symptoms associated with EAE. Severity of symptoms were used to grade EAE as follows: Grade 0, asymptomatic; Grade 1, weak or hooked tail; Grade 2, floppy tail indicating complete loss of tonus in tail; Grade 3, floppy tail and hind limb paresis (splaying of limbs, slow or unsteady gait, hind limbs slip off the bars while walking on the lids of the cages), Grade 4: floppy tail and unilateral hind limb paralysis; Grade 5, floppy tail and bilateral hind limb paralysis. Animals were killed as the disease peaked, determined by stabilization of the grade for 2 or more days, or when they attained the ethically permitted limit of grade 5. Mice were deeply anaesthetized and perfused intracardially with ice-cold Phosphate Buffered Saline (PBS), and spinal cords were dissected out.

### Flow cytometry

Spinal cords were collected in ice cold Hanks Balanced Salt Solution (HBSS) (Invitrogen A/S, Taastrup, Denmark). Cell suspensions were prepared by mechanical dissociation and forcing through a 70 mm cell strainer (BD Biosciences, Brøndby, Denmark). Myelin in the samples was removed following centrifugation on 37% isotonic Percoll (GE Healthcare Bio-sciences AB, Uppsala, Sweden).

T cells were stimulated for 9 h in 96 well plates coated with anti-mouse CD3ε (clone 145-2C11) in the presence of 1 μl/ml GolgiPlug (BD Biosciences) that was added 2 h after plating, to trap the cytokines within the cells.

The cells were washed and stained with PerCP/Cy5-CD8 (clone 53–6.7), FITC-CD4 (clone GK1.5) or V500-CD4 (clone RM4-5) (BD Biosciences, Brøndby, Denmark), APC- or PE-CCR6 (clone 29-2L17) (Biolegend), PE-IL-17 (clone TC11-18H10.1) (Biolegend), PE-Cy7-IFNγ (clone XMG1.2) (Biolegend) and biotinylated CXCR3 (clone CXCR3-183) (Biolegend) detected using APC- or PE-streptavidin. Individual isotype controls were performed for each sample. Data was collected on LSRII (BD Biosciences, San Jose, CA) and analyzed using FACS DIVA (BD Biosciences) and FlowJo software (Tree Star, Ashland, OR).

For sorting CCR6 expressing T cells in the CNS, cells were stained with PerCP/Cy5-CD8 (clone 53–6.7), FITC-CD4 (clone GK1.5) and PE-CCR6 (clone 29-2L17). Cells were sorted on a FACSVantage/Diva cell sorter (BD Biosciences) from pooled batches of CNS isolates from 5 mice with MOG p35-55-induced EAE. The experiment was repeated twice to generate three replicate samples of T cells isolated from CNS, from separate EAE inductions.

### Double immunohistochemistry

Spinal cords were dissected out from PBS perfused mice, placed in 4% paraformaldehyde (PFA, Sigma-Aldrich, Denmark) in PBS, then immersed in 30% Sucrose and frozen as described previously (Mony et al., 2014). Spinal cord sections (16 μm thick) were cut on a cryostat and stored at −80°C. In brief, sections were postfixed in 4% PFA, and after several washes in PBS and PBS containing 0.2% Triton-X100 (PBST), they were then incubated with blocking solution containing 3% Bovine serum albumin in PBST. Sections were stained with PE-CCR6 (clone 29-2L17, Biolegend) and FITC-CD4 (clone GK1.5). Nuclei were stained using 4′,6-diamidino-2-phenylindole (DAPI) (Invitrogen-Molecular Probes). Isotype-matched primary antibodies were used to control for non-specific staining. Images for CCR6 expression in CD4^+^ T cells were acquired using an Olympus BX51 microscope (Olympus, Denmark) connected to an Olympus DP71 digital camera, and combined using Adobe Photoshop CS version 8.0 to visualize double-labeled cells.

### Quantitative real-time PCR

RNA was extracted from sorted cells according to the manufacturer’s protocol for TRIzol (Invitrogen Life Technologies). Moloney murine leukemia virus RT (Invitrogen Life Technologies) was used to synthesize cDNA from the total RNA using random hexamer primers. Quantitative Real-Time Reverse Transcriptase- PCR assays (qRT-PCR) for IFN-γ, IL-17, T-bet, ROR-γt, and 18S rRNA (Applied Biosystems) were performed using ABI Prism 7300 Sequence Detection Systems (Applied Biosystems, Foster City, CA). The following primer and probe sequences were used: IFN-γ (Forward CATTGAAAGCCTAGA AAGTCTGAATAAC, Reverse TGGCTCTGCAGGATTTTCATG, Probe TCACCATCCTTTTGCCAGTTCCTCCAGMGB), IL-17 (Forward CTCCAGAAGGCCCTCAGACTAC, Reverse TGTGGT GGTCCAGCTTTCC, Probe ACTCTCCACCGCAATGAMGB), ROR-γt (Forward CCGCTGAGAGGGCTTCAC, Reverse TGCA GGAGTAGGCCACATTACA, Probe AAGGGCTTCTTCCGCC GCAGCCAGCAG TAMRA). The expression of T-bet and GM-CSF was determined using Mm01299452-g1- and Mm00438328-m1 TaqMan gene expression assays (Applied Biosystem), respectively. Relative RNA levels in the samples were determined using standard curves prepared from four-fold serial dilutions of cDNA from a reference sample. Relative expression levels of genes were normalized to 18S rRNA in the samples.

### Statistical analysis

Data was analyzed using GraphPad Prism version 6.0 (GraphPad Software Inc., San Diego, California, USA). CCR6 expression was analyzed using nonparametric Mann-Whitney *t*-test. CCR6 expression in IFNγ, IL-17 and IFNγ+IL-17+ CD4+ T cells was analyzed by one-way ANOVA. Values of *p* < 0.05 were considered statistically significant.

## Results

**Figure 1:**
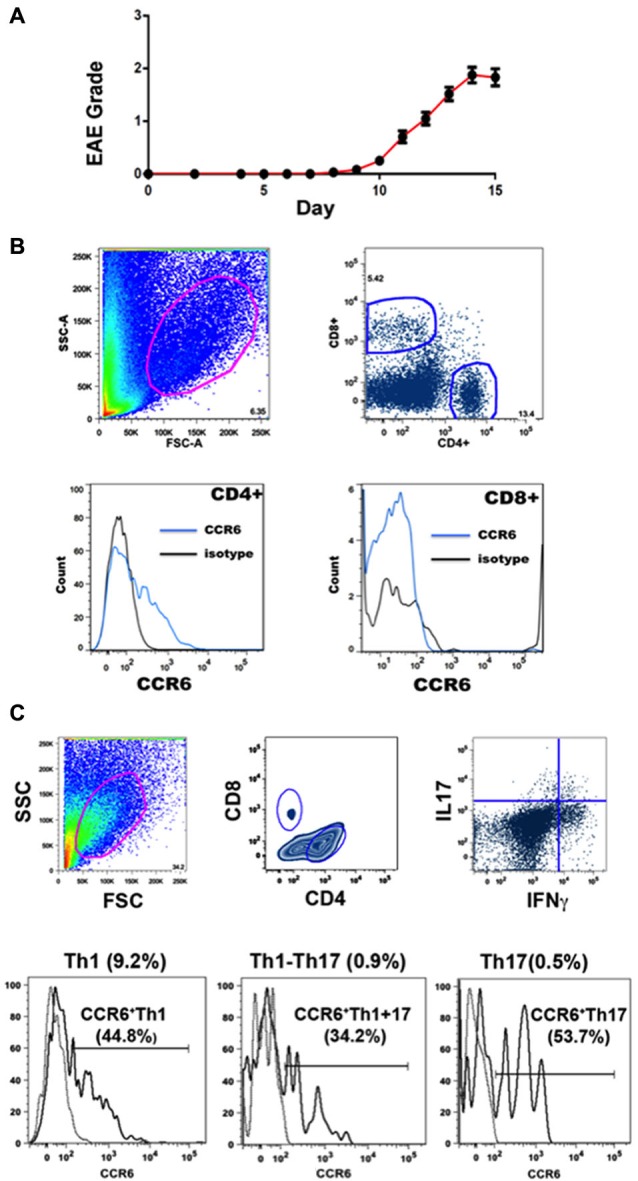
**EAE and flow cytometry. (A)** Progression of EAE in mice immunized with MOGp35-55 (Mean clinical scores ± SEM, *n* = 184). **(B)** Gating strategies for flow cytometry analysis. CCR6 expression was detected in CD4^+^ T cells but not CD8^+^ T cells in CNS. Cells in the spinal cord were gated on the basis of size and granularity (top left), followed by CD4 and CD8 expression (top right) and CCR6 expression (CD4^+^, lower left) and (CD8^+^, lower right). Each sample was individually controlled for binding of isotype-matched antibody of irrelevant specificity. **(C)** Gating strategy for cell sorting. CCR6 expression was detected in both IL17 and IFNγ producing CD4^+^ T cells in spinal cord. Representative gating scheme depicting the morphological gate (top left), CD4 and CD8 gates (top middle), and IFNγ vs IL17 expression in CD4^+^ T cells. Subsequently, IFNγ, IL17 and IFNγ-IL17 dual producers were gated reative to isotype control for CCR6 expression (bottom histograms). Proportions of each subset within CNS CD4^+^ isolates are shown on top of each profile, and the percentages that expressed CCR6 are shown with the histograms.

EAE was induced by immunization with MOGp35-55, a commonly used encephalitogen (Gold et al., 2006). Onset of disease was usually at about day 10 and progression showed a rapid increase in clinical score that levelled off after a few days. Our definition of peak disease was two sequential days where clinical score did not increase, at which point mice were sacrificed for molecular and histological analyses. Figure 1A shows the disease course for the animals in this study.

Infiltrating lymphocytes and leukocytes were analyzed by flow cytometry. Gating strategies are shown in Figure 1B. Characteristically for EAE, populations were quite heterogeneous, including TCR β^+^ T cells and CD11b^+^ myeloid cells (macrophages, neutrophils and dendritic cells) (Zehntner et al., 2005; Gold et al., 2006; Toft-Hansen et al., 2011). The majority (78.9 ± 2.3%, *n* = 10) of T cells were CD4^+^. Expression of CCR6 by CD4^+^ and CD8^+^ T cells was analyzed by flow cytometry. Whereas a large proportion (15.9 ± 7.5%, *n* = 23) of CD4^+^ T cells expressed CCR6, almost no CD8^+^ T cells expressed this receptor (Figure 1B). We have described elsewhere that the majority of CD8^+^ T cells expressed CXCR3, which was variably expressed by CD4^+^ T cells (Mony et al., 2014). We did not directly assess whether individual T cells expressed both chemokine receptors.

**Figure 2:**
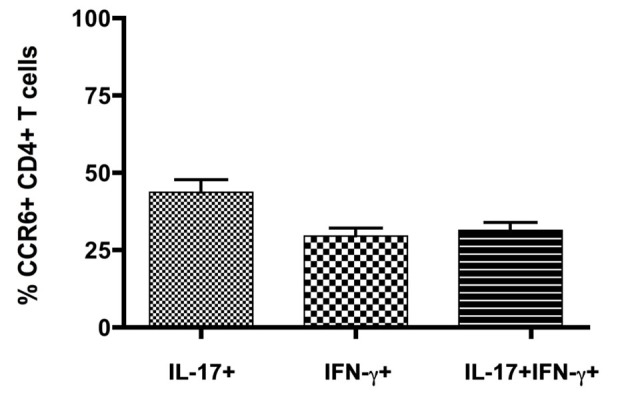
**CCR6 expression by IFNγ- and IL17-producing CD4^+^ T cells in the CNS**. Cytokine producing T cells isolated from CNS of mice with severe EAE were detected by flow cytometry following *in vitro* restimulation and intracellular cytokine staining. CCR6 expression was analyzed on cells gated for CD4 and intracellular IL-17 and IFNγ, relative to isotype control. Bars show means ± SEM (*n* = 8).

Expression of inflammatory cytokines was measured by flow cytometric intracellular cytokine staining. Th1 IFNγ-producing CD4^+^ T cells (6.8 ± 0.7%, *n* = 8) greatly outnumbered other subsets, and together with those that produced both IFNγ and IL-17 (Th1+17) (0.9 ± 0.2%, *n* = 8), cells producing IFNγ constituted over 90% of cytokine-producing CD4^+^ T cells in the CNS (Figure 1C) (also see Mony et al., 2014). Th17 IL-17-producing CD4^+^ T cells constituted 0.7 ± 0.1% (*n* = 8) of the total. CD8^+^ T cells produced IFNγ but did not produce IL-17 to any significant extent (Mony et al., 2014). Notably, CCR6 was expressed by 30–60% of CD4^+^ T cells in intracellular cytokine assays, regardless of their cytokine profiles (Figure 1C). There was no significant bias towards or against CCR6 expression by Th1, Th17 or Th1+17 subsets (Figures 1C, 2).

**Figure 3:**
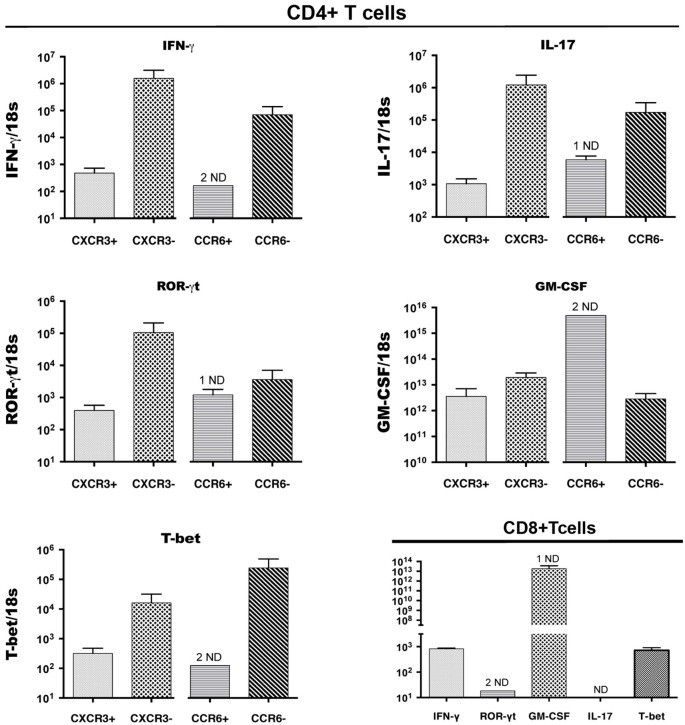
**Cytokine and transcription factor (TF) gene expression by CCR6^+^CD4^+^, CXCR3^+^CD4^+^ and CD8^+^ T cells in the CNS**. CD4^+^ T cells expressing CXCR3 or CCR6, and CD8^+^ T cells, were sorted from spinal cords of mice with EAE, pooled from groups of 5 mice, in three different experiments. IFNγ, IL-17, GM-CSF, RORγt, T-bet mRNA expression was detected byQRT-PCR in CXCR3-expressing and CCR6-expressing, as well as receptor-negative CD4^+^ T cells from the same sorts (Top 4 and bottom left panels). Bottom right panel: CD8^+^ T cells in the CNS expressed IFNγ, GM-CSF, RORγt, Tbet mRNA, but not IL-17. The y axis shows relative levels of expression (compared to a standard curve) as a ratio to 18S rRNA levels in the same sample. ND: not detectable (1 indicates 1 sample only ND).

Expression of cytokines and of transcription factors that control expression of key cytokines was also examined byQRT-PCR analysis of cDNA from CD4^+^ T cell populations that were sorted on the basis of CCR6 and CXCR3 expression from CNS infiltrates of mice with peak EAE. Figure 3 shows that, as for intracellular cytokines, there was no significant bias towards or against expression of IFNγ or IL-17 message on the basis of surface expression of either of these chemokine receptors. This was also true for GM-CSF, a cytokine that has been implicated as a direct encephalitogenic mediator in EAE (Kroenke et al., 2010; Codarri et al., 2011). Similarly, no bias was seen for expression of Tbet and RORγt, the transcription factors that control expression of IFNγ and IL-17, respectively. Lack of detectable signal in some of the sorted populations of CD4^+^ CCR6^+^ T cells likely reflects low amounts of RNA in those samples. Populations sorted on the basis of lack of expression of either CCR6 or CXCR3 showed equivalent if not greater levels of message for all cytokines and transcription factors as those sorted for chemokine receptor expression, although populations identified on the basis of lack of expression of a single receptor are intrinsically less informative. CD8^+^ T cells sorted from the CNS of mice with peak EAE contained equivalent levels of mRNA for both IFNγ and GM-CSF to those in CD4^+^ cells, but did not express detectable IL-17 message (Figure 3).

**Figure 4:**
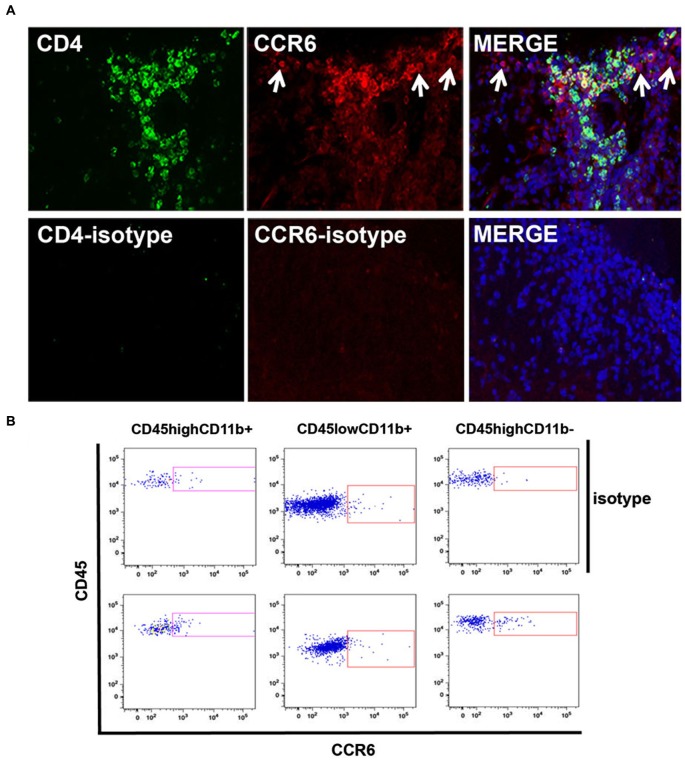
**Cellular localization of CCR6 expression in CNS. (A)** CCR6 expression colocalized with CD4^+^ T cells in spinal cord infiltrates in EAE. CCR6 expression was detected on CD4^+^ cells by immunofluorescence microscopy in subpial infiltrates in EAE spinal cord. CD4^+^ cells that lacked CCR6 expression can also be seen. CCR6 expression was also detected in cells that lacked CD4 expression (arrows). Micrographs are representative of tissue from 4 mice. Non-specific staining was evaluated by replacing CD4 and CCR6 antibodies with isotype-matched negative control antibodies. **(B)** Flow cytometry analysis of CCR6 expression by CD45^high^CD11b^+^, CD45^low^CD11b^+^ and CD45^high^CD11b^-^ cells in spinal cord of MOGp35-55-immunized mice with EAE. Profiles show CD45 and CCR6 (bottom panels) or isotype control (top panels) staining on cells gated by relative CD11b expression.

We then localized CCR6-expressing cells within infiltrates by immunofluorescence microscopy. Figure 4 shows that CCR6^+^ cells were numerous within infiltrates in spinal cord of mice with peak disease, and that many of them co-expressed CD4. These are included within the CD4^+^ CCR6^+^ cells that were sorted and analyzed by flow cytometry. There were also a significant number of CD4^+^ cells that did not express CCR6, which may be assumed to include CXCR3^+^ CD4^+^ T cells. CCR6^+^ cells that did not express CD4 were also observed (arrows). Staining with antibody against GFAP and morphology excluded that these were astrocytes (not shown). For technical reasons it was difficult to co-localize CCR6 with myeloid markers in tissue sections, so we used flow cytometry to determine whether CD11b^+^ cells also expressed CCR6. Those data are shown in Figure 4B. In two separate analyses we could show an increased proportion of CCR6-expressing CD11b^+^CD45^high^ (eg infiltrating, blood-derived) cells. Almost no CD11b^+^ CD45^dim^ microglia from the same isolates could be shown to express CCR6, although we cannot exclude that a few of these cells were CCR6^+^—neither of these populations were further examined or localized. As expected, there was a significant proportion of cells expressing CCR6 within the CD45^high^ CD11b-negative population, which include infiltrating T cells. Flow cytometry confirmed that CD8^+^ cells in CNS did not express CCR6 (not shown).

Thus, both CD4^+^ T cells and macrophages expressed the CCR6 chemokine receptor in spinal cord infiltrates of mice with EAE.

## Discussion

Interplay between CNS- and immune-derived signals is central to induction and regulation of neuroinflammatory diseases such as MS. The possibility that chemokines might selectively recruit T cells with distinct functional capability opens scenarios that are both of fundamental interest as well as offering therapeutic options. We have asked whether T cells that were recruited to the CNS of mice with EAE show selective expression of the CCR6 chemokine receptor, that had been identified as aligning with the IL-17-producing CD4^+^ Th17 cytokine subset in studies of experimentally polarized T cells. A previous study had addressed this by taking a post-hoc approach of measuring Th subsets that had already infiltrated to induce severe EAE, and determining their chemokine receptor expression, but had not examined CCR6 or Th17 within CNS infiltrates (Fife et al., 2001). Taking a similar approach we demonstrate, as far as we know for the first time, that the Th1 and Th1+17 subsets, both producing IFNγ, overwhelmingly predominated in CNS, and that many Th1 as well as Th1+17 expressed CCR6. We also find that almost no CD8^+^ T cells in CNS expressed CCR6 or IL-17, but were overwhelmingly IFNγ-producers that expressed CXCR3. Both CD4^+^ and CD8^+^ T cells expressed GM-CSF, and expression of the Th1 and Th17-associated transcription factors T-bet and RORγt aligned with IFNγ and IL-17 respectively.

These findings support three broad interpretative conclusions: (1) CCR6 can be expressed by Th1 and by Th1+17 as well as by Th17 in CNS; (2) IFNγ-producing T cells are a major component of the neuroinflammatory response in EAE; and (3) The spectrum of chemokines and their receptors that control immune infiltration to the CNS is likely to be quite broad. These will be discussed in turn.

CCR6 is the chemokine receptor for CCL20 (liver activation regulated chemokine, LARC or macrophage inflammatory protein-3α, MIP3α) (Baba et al., 1997; Greaves et al., 1997; Hieshima et al., 1997; Rossi and Zlotnik, 2000). The constitutive expression of CCL20 in choroid plexus is proposed to act as a gateway for T cells into uninflamed CNS (Axtell and Steinman, 2009; Reboldi et al., 2009). CCL20 and CCR6 expression are upregulated in the spinal cord in EAE (Serafini et al., 2000). CCL20 is expressed mainly by leukocytes infiltrating the CNS of SJL mice at the onset (acute phase) of relapsing-remitting EAE. CCL20 is also expressed in astrocytes after disease relapses (chronic phase) in the SJL/J EAE model (Serafini et al., 2000; Ambrosini et al., 2003). The cytokines IL1β, IL6, TNFα and combinations of IL1β and TNFα, IL6 and IL-17 can induce CCL20 in astrocyte cultures, whereas IFNγ and IL-17 do not (Ambrosini et al., 2003; Kang et al., 2010; Meares et al., 2012). IL1β, IL6 and TNFα expression are elevated in the brains of mice before the onset of symptoms in EAE (Murphy et al., 2010). IL-17 and downstream Act1 signaling enhanced TNF α-induced CCL20 expression in astrocytes (Kang et al., 2010), which could facilitate the entry of CCR6 expressing T cells into the CNS.

The leukocytic infiltrate in EAE is heterogeneous and includes, as well as T cells that are not specific for the disease-inducing immunogen MOG, macrophages, neutrophils and DC. CCR6 is expressed by many cell types, including B cells, T cells, DC, neutrophils and macrophages (Wojkowska et al., 2014 and reviewed in Lee et al., 2013). Th17 and regulatory T cells have come under the spotlight as CCR6^+^ cells that play an important role in MS and EAE (Reboldi et al., 2009). Identification of CD4^+^ T cells which do not express CCR6 in an inflammatory context is therefore of interest. A defining characteristic of chemokine immunology is redundancy, so suggestion that a particular receptor or chemokine ligand would not be essential might not seem all that informative. However, the CCR6-CCL20 receptor-ligand pair is unusual in being non-redundant so neither can be substituted by other receptors or ligands, in the context of the paired interaction. Whether other receptor-ligand pairs can substitute for functional outcome then becomes a question. A recent study showed that CNS-infiltrating Th17 expressed CXCR2 (Wojkowska et al., 2014). Whether there is an absolute requirement for CCR6 for Th17 entry to the CNS has not been resolved.

One potential issue for interpretation of chemokine receptor analyses is that receptor ligation by chemokine may have led to downregulation of the receptor. We cannot exclude that this may have occurred and that the actual proportion of CNS-infiltrating CCR6^+^ Th17 may have been higher than we estimated. However, since relatively low but comparable (>25%, <50%) proportions of any cytokine subset expressed CCR6, this argues against all of these T cells depending on CCR6 for their entry to the CNS, as well as against subset-specific dependence. Furthermore we show no CCR6^+^ CD8^+^ T cells, although all of them had infiltrated, and in this and another study we have shown that all of the CD8^+^ and significant proportions of CD4^+^ (of any cytokine subset) express CXCR3. We did not pursue the role of CXCR3^+^ T cells further, and studies of the role of CXCR3^+^ T cells in EAE continue to yield quite divergent findings (Liu et al., 2005; Muller et al., 2010; Sporici and Issekutz, 2010; Lalor and Segal, 2013). Our data does not exclude that CCR6-negative T cells had once expressed CCR6. Despite the potential for downregulation of CCR6 expression by T cells following encounter with CCL20, we show that Th1 as well as mixed Th1+Th17 do express CCR6.

There is a divergent literature on the role of CCR6 in EAE. Adoptive transfers showed that CCL20 was not required for the effector phase of EAE, although neutralizing antibodies reduced disease severity (Kohler et al., 2003). Mice deficient in CCR6 or treated with blocking antibodies, although relatively resistant to EAE, nevertheless developed mild disease (Liston et al., 2009; Reboldi et al., 2009; Moriguchi et al., 2013). Other studies showed that mice lacking CCR6 actually developed more severe or chronic EAE, attributed either to reduced regulatory T cell recruitment (Villares et al., 2009), or lack of CCR6^+^ PDL1^+^ mDC (Elhofy et al., 2009). In all of the knockout studies, CCR6-deficient T cells infiltrated the CNS.

The predominance of IFNγ-secreting T cells in the CNS of mice with severe EAE is very striking. There have been conflicting reports on the role and requirement for IFNγ in EAE. This is the only cytokine to have been directly shown to be pro-pathogenic in MS (Panitch et al., 1987), although that is not necessarily a desirable or easily achievable demonstration for other cytokines. Recent papers have provided a more nuanced perspective on the role for IFNγ in EAE and MS, showing that timing and possibly location of expression influence outcome of its expression (Hindinger et al., 2012; Naves et al., 2013). The mixed Th1+17 subset is a prominent and consistent feature of our analyses of MOG-induced EAE and has been implicated in MS (Kebir et al., 2009). It has been reported that polarized Th17 can convert to IFNγ-producing T cells *in vivo* (Shi et al., 2008; Bending et al., 2009; Lee et al., 2009). One of the roles recently identified for IFNγ is controling recruitment of Th17 (Berghmans et al., 2011), which increases interest in the Th1+17 subset. The previously bipolar debate on the relative roles of Th1 versus Th17 in EAE is given broader perspective by such considerations. Also, it is now clear that neither of the nominal cytokines for Th1 or Th17 are themselves necessary for EAE, but a third cytokine GM-CSF plays a key role (Kroenke et al., 2010; Codarri et al., 2011). We show that this cytokine is produced by CD4^+^ and CD8^+^ T cells and that as for IFNγ and IL-17, there is no obvious correlation with expression of the CCR6 or CXCR3 chemokine receptors. Our study has not attempted to differentiate between whether these cytokines are necessary or sufficient for disease, but does not support that the CCR6 chemokine receptor aligns with any of them. Expression by macrophages points to the possibility of their interaction with CCL20-producing astrocytes, an aspect that deserves further attention. It cannot be excluded that some microglia may also express CCR6.

The importance of CCR6 signaling for induction of EAE is shown by disease reduction in mice lacking this receptor, and by studies in which the receptor or its CCL20 ligand were blocked (Kohler et al., 2003; Liston et al., 2009; Reboldi et al., 2009). A question that arises is whether this receptor selectively controls entry of Th17 to the CNS. Findings from direct analysis of T cells that had entered the CNS in established EAE do not support this, nor do they support any association with GM-CSF producing cells. Similarly this and another study do not support association of CXCR3 with Th1 or IFNγ-producing T cells (Mony et al., 2014). Importantly our analyses also identify T cells that may not express either of these chemokine receptors. This points to there being a wider spectrum of chemokine responses driving EAE rather than only CCR6 and CXCR3. One candidate pathway involves CCR2, which has three potential ligands, although it is more implicated in regulation of macrophage entry. Other receptors such as CCR8 have also been implicated, especially in TNF-driven induction of glial response at the blood-brain barrier (Mürphy et al., 2002), as well as CXCR2 (Wojkowska et al., 2014). It has been reported that Th17 can co-express CCR4 and CCR6 (Mehling et al., 2010), but EAE (with reduced severity) could be induced in a double knockout mouse, and CCR6-negative CD4^+^ T cells infiltrated the CNS (Moriguchi et al., 2013). The possibility of substitution by other receptor-ligand interactions might help explain lack of black-vs-white findings from ablation or blockade of selected chemokines or their receptors.

We have used direct analyses to show lack of alignment between chemokine receptors with T cell cytokine subsets in the inflamed CNS. This highlights challenges for development of chemokine-directed therapy for MS, and underlines the elegance, complexity and tremendous importance of chemokines in controling immunesurveillance as well as pathophysiologic T cell entry to the CNS.

## Authors and contributors

Jyothi Thyagabhavan Mony and Reza Khorooshi: design and planning, acquisition analysis and interpretation of data, drafting and revising manuscript, approving manuscript, accountability for accuracy and integrity. Trevor Owens: design and planning, analysis and interpretation of data, drafting and revising manuscript, approving manuscript, accountability for accuracy and integrity.

## Conflict of interest statement

The authors declare that the research was conducted in the absence of any commercial or financial relationships that could be construed as a potential conflict of interest.
